# From content to context: A qualitative case study of factors influencing audience perception of the trustworthiness of COVID-19 data visualisations in UK newspaper coverage

**DOI:** 10.1177/14648849231190725

**Published:** 2023-07-27

**Authors:** Jingrong Tong

**Affiliations:** 7315 The University of Sheffield, UK

**Keywords:** Audience perception, news trustworthiness, COVID-19 data visualisations, UK newspaper coverage, data, context, social construction

## Abstract

Drawing from 18 audience interviews, this article examines audience perception of the trustworthiness of COVID-19 data visualisations in UK newspaper coverage. The findings suggest that overall, the participants viewed the selected COVID-19 data visualisations as largely trustworthy. Their perception was unaffected by the types of data visualisations. The trustworthiness of data visualisations had no clear connection with their likability and learnability. Instead, the participants’ trust was influenced by the perceived problematic presentation of data visualisations, such as the inappropriate use of bars to represent data or the failure to present data in context. It was also affected by the participants’ understanding of the problems about data (production and presentation), their assessment of the credibility of data sources and news outlets, and their personal lived experiences and information gained from other sources. All of these were related to the social context surrounding data and data visualisations, rather than merely the content of data visualisations. The findings reveal that the social construction nature of data and data visualisations creates a space for the participants to question data visualisations’ trustworthiness. The close connection between trust in data visualisations and trust in data, a socially constructed product, suggests that the trustworthiness of data visualisations transcends the control of journalists and news media, extending to the context of data and its visualisations. This qualitative research reveals the importance of context to audience trust in data visualisations in the UK.

## Introduction

Data visualisations have become increasingly prevalent in news coverage (Kennedy and Engebretsen, 2020), particularly since the onset of the COVID-19 pandemic. However, research on the reception of data visualisation is scarce (Engebretsen, 2020), leaving us with limited knowledge of how audiences perceive data visualisations in general and, in particular, their trustworthiness.

This study fills the gap in the literature. In this study, 18 interviews were conducted with UK audiences. Participants were asked to evaluate the trustworthiness, likability and learnability of selected COVID-19 data visualisations published in the news without the presence of the accompanying text. This study contributes to the literature on audience trust in news and data visualisations.

This article first discusses the literature on audience reception and trust in news. It then introduces the present study, including its process, method and data. A discussion of the findings will be presented in the next sections, followed by a reflection on the limitations of this study.

## Theoretical framework: Audience reception, trust in news and data visualisations

Trust is crucial for the proper function of a society and “brings us all sorts of good things” (Uslaner, 2002: 1). For communication research, trust is considered a vital parameter informing us of how audiences perceive and assess news media and their content. In the literature, terms including “trust”, “trustworthiness” and “credibility” are used interchangeably (Kohring and Matthes, 2007).

Trust in news (media) plays a crucial role in audience acceptance and consumption of news media as well as fostering civic participation. However, it has been reportedly declining in societies like the United Kingdom (UK) over the past decades, albeit with variations across countries (Park et al., 2020). Despite the various changes induced by the COVID-19 pandemic, the issue of low trust in news persists as an overarching concern. Trust in news initially declined during the early stages of the pandemic (Newman et al., 2020), followed by a subsequent recovery (Newman et al., 2021), but ultimately experienced another downturn after COVID-19 bumps ended (Newman et al., 2022).

The studies examining trust in news (media) started in the 1970s and have burgeoned since the 1990s (such as McCroskey and Jenson, 1975; O'Keefe, 1990; Kohut and Toth, 1998; Austin and Pinkleton, 1999). During the pre-digital era, including the 1990s and before, scholars (such as McCroskey and Jenson, 1975; Austin and Pinkleton, 1999) focused on examining traditional news media. Later, they (such as Cassidy, 2007; Tsfati, 2010) extended their interest to audience trust in online news (sites). Earlier studies focused on the correlation between media effects (or agenda setting) and audience trust in news (media) and its impact on news media’s democratic roles (such as Tsfati, 2002; Tsfati, 2003; Kiousis, 2001; Tsfati and Cappella, 2003; Tsfati and Cappella, 2005). Along with the emergence of the idea of active audiences, a large body of the literature has gradually shifted from examining media effects and trust to probing how audiences evaluate news trustworthiness and the factors that influence audience (perception of) trust in news (media) (such as Lee, 2010; Amazeen and Muddiman, 2018; Swart and Broersma, 2022). Measures such as reducing journalistic bias and opinion in the news are seen to boost trust in the news (Fisher et al., 2021; Henke et al., 2020).

The literature sees trust as complex and multiple-dimensional (Horowitz et al., 2021; Kohring and Matthes, 2007). Trust in news (media) is defined as trust in journalistic selections of topics, facts and journalistic depictions and assessments (Kohring and Matthes, 2007). It refers to the news sources cited in the news, the message conveyed within the news, and the news media responsible for its publication. It can be summarised as perceived “source credibility”, “message credibility” and “media or medium credibility”. Source credibility is defined as “judgments made by a perceiver concerning the believability of a communicator”, regarding a communicator’s expertise and trustworthiness (Hovland et al., 1953; O'Keefe, 1990: pp. 181). Message credibility has been examined in terms of “message structure”, “message content”, “language intensity” and “message delivery”. Media/medium credibility research typically focuses on the types of news media through which the message is published, such as newspapers or television (Golan, 2010). The literature also considers trust in news as a fundamental component of public trust in the government and the political establishment (Marcinkowski and Christopher, 2018). It can be influenced by audiences’ partisanship, political ideology, and political and personal cynicism (Lee, 2010). This means that media trust can also be influenced by external factors such as political trust or the legitimacy of social institutions.

Since the COVID-19 pandemic emerged, news outlets around the world have embarked on an unprecedented utilisation of data and data visualisations within their news coverage. When it comes to news that uses data and data visualisations, the literature highlights the difference made by numbers—statistical data—and data visualisations to audience trust in news. Overall, two contrasting arguments exist regarding the association between the incorporation of numeracy and data visualisations in news stories and how the audience perceives their trustworthiness. One view (such as the articles in Nguyen, 2018; Porter, 1995; Van Dijk, 1988: 87) generally sees the use of numbers and data visualisations in news generating trust (Beer, 2016; Lindsey and Yun., 2003; Koetsenruijter, 2011). The other view questions the effect of using numbers in news. A cross-country study found that news stories with numbers were not appealing to audiences in the United States, Zambia and Tanzania. Audiences with lower levels of numeracy appeared to trust news stories with numbers more than those with a better understanding of numeracy (Gondwe et al., 2021). Prior knowledge and issue involvement influence audiences’ ability and incentive to process information (Lee and Kim, 2016), with those who have prior knowledge finding it easier to process information. These discussions highlights the importance of audiences’ backgrounds in shaping their perception of the trustworthiness of the news that uses numbers and data visualisations.

In addition, Henke and collaborators found that the inclusion of statistical information and data visualisations in the news can contribute to enhancing audience perception of news trustworthiness. However, having too much statistical data in the news may lead to a cognitive burden and make it difficult for audiences to understand (Lee and Kim, 2016; Henke, et al., 2020). Also, news stories using numbers often have, or are believed to have, errors and misleading information (Maier, 2002), leading to mistrust in the news media (Peters, 2020).

These discussions in the literature point to four important aspects of trust in news: (1) trust in news is multidimensional, shown as trust in news sources, news content and news media; (2) trust in news can be influenced by external factors; (3) the utilisation of numbers and data visualisations in news can earn the trust of audiences, but it also depends on the numeracy levels of the audience and the quality of the information provided and may result in other issues such as information overload; (4) prior knowledge and motivation to process information are important to audience perception of the trustworthiness of news using numbers and data visualisations.

When it comes to audience trust in data visualisations, current studies have mostly seen data as numbers but neglected to consider the influence of its social construction nature on shaping audiences’ perceived trust in news. Data extends beyond mere numbers; it encompasses any information or content that can be digitized. Data is inherently socially constructed and intertwined with social dynamics and power relations within the context in which it is collected and produced (Jenkins, 2019). Audience perception of trust in data visualisations can be influenced by the audience’s comprehension of data and the manner in which data is presented visually. Since our understanding in this area is limited, further research is thus necessary.

The existing studies have also largely examined data visualisations as an integral component of the news. This approach has its advantages, as in real-world scenarios, data visualisations often accompany news articles, allowing audiences to consume them simultaneously. However, in this approach, the combination of visuals and texts, being two different systems, has the potential to amplify and influence their respective meanings (Bateman, 2014). The perception of data visualisations by the audience can be influenced by the accompanying textual content of news articles. Data visualisations are standalone cultural artefacts, containing data and visual representations of data (Rettberg, 2020). The visuality of data visualisations possesses a communicative and persuasive power that differs from, and often surpasses, that of text. It has the ability to evoke users’ emotions by bypassing rational judgment (Kennedy and Hill, 2018; Fratczak, 2022). It can also engender an uncritical trust in numbers, thereby contributing to the restoration of public trust (Beer, 2016; Sleigh and Vayena, 2021; Nærland and Engebretsen, 2023). To understand audiences’ perception of data visualizations solely based on their inherent qualities, independent of the textual form of the news, it is necessary to examine data visualizations outside the context of news articles. However, it is important to note that the absence of the actual reading context of data visualizations might hinder audiences’ perception of them. This is because certain information that is obscure or missing in data visualisaitons may be included in the text of news, and the understanding gained from reading the text can enhance the audience’ interpretation of data visualisations. But the advantage of omitting the text of news lies in the ability to analyze data visualizations independently. This allows us to understand how the presentation of data visualizations influences audiences’ trust and perception, without being influenced (or distracted) by the textual content of news articles. This approach also helps us explore whether certain types of data visualizations evoke stronger perceptions of trustworthiness and to what extent. These aspects are, however, understudied in the current literature and related studies are scarce (Kennedy and Engebretsen, 2020; Engebretsen, 2020). Scholars (such as Henke, et al., 2020; Lee and Kim, 2016) called for more research about the perception of data visualisation.

## The study

This research will address these aspects by interviewing 18 participants about their perception of the trustworthiness of COVID-19 data visualisations published in the news coverage by UK news media. COVID-19 data visualisations were abundant in news coverage, both in the UK and beyond, serving as a significant tool for communicating pandemic-related information to the public. Therefore, it is important to understand how audiences perceive the trustworthiness of COVID-19 data visualisations since this aspect has not yet been thoroughly researched. However, it is expected that audiences would have a higher level of familiarity with COVID-19 data visualisations and the associated data compared to those related to other topics. While this familiarity can facilitate participants’ discussions during the interviews, it also has the potential to influence the findings of the present study. In addition, the pandemic has also triggered a wide range of controversial issues such as social distancing, lockdowns, true death numbers, and vaccines. While reading data visualisations on these topics, the political and ideological stances, as well as personal experiences of the audience can potentially impact their perception of these data visualisations’ trustworthiness.

The semi-structured interviews took place in 2021, a time the UK was on the verge of re-emerging from its third national lockdown and the safety issue surrounding AstraZeneca was in spotlight. The participants (see Table 1 for their backgrounds) were recruited through snowball sampling and the internet, particularly Twitter and Facebook (now Meta). Table 1 suggests these participants were well-educated, and most were in well-paid professions. 15 out of 18 participants were under 50. Interviews lasted between one and 3 hours. 11 COVID-19 data visualisations published by UK news media, including *The Guardian*, *The Times*, *The Financial Times, The Daily Telegraph*, *The Independent*, *The Daily Mail* and *The Economist,* were selected before the interviews, according to their types and themes. 10 data visualisations were published by quality newspapers, with only one published by *The Daily Mail*, a tabloid. They cover key topics related to the COVID-19 pandemic including social distancing, lockdowns, the economic impact, case and death numbers, as well as blood clot concerns and the AstraZeneca vaccine.

**Table 1.** Interview participants’ personal backgrounds.

Career	
Undergraduate student	3
IT industry	3
Education	2
Medicine	2
Media	2
Marketing	2
Masters’ student	1
Social worker	1
Data analyst	1
Administration	1
Age	
20–29	4
30–39	1
40–49	10
50–59	2
60–69	1
Education	
University	5
PhD	4
Masters	4
Diploma	1
University student	3
Masters’ student	1

Each participant was asked to view the data visualisations individually and explain their thoughts. The data visualisations were presented independently without the accompanying text of the news articles. The advantage of this research design is that data visualisations can be examined as standalone visual cultural artefacts and audiences’ perception of them will not be influenced or distracted by the accompanying text. However, removing the immediate reading context of these data visualisations has a downside, as it may limit the participants’ understanding of their meanings and contextual backgrounds. As previously discussed, data visualisations and the textual content of news articles can mutually influence their respective meanings. Their interdependence means if audiences struggle to understand data visualisations, they may turn to the text of news articles for answers. This is because some information may be included in the text of news articles rather than data visualisations themselves, and the textual content can aid audiences in interpreting the meaning of the data visualisaitons. The analysis and conclusion of this research took this limitation into account. Of the 18 participants, one read and discussed seven data visualisations and another eight. The other 16 did 11. Three main aspects of the data visualisations were discussed: trustworthiness, likability and learnability. The participants were asked to rate these data visualisations for these aspects between 0 (not trustworthy) and 5 (trustworthy).

Interviews were recorded and transcribed verbatim after permission was received from the participants. The interview transcripts were uploaded and analysed in Nvivo qualitatively. Thematic analysis (Braun and Clarke, 2006) was conducted, and the transcript text was explored repeatedly to allow themes to emerge. The research only kept the themes with adequate quotes across all or most transcripts. The analysis of their perceived trustworthiness focused on how the participants perceived the trustworthiness of the data visualisations and for what reasons. Their discussions about the likability and learnability of data visualisations were also considered in the analysis. The names used in this article are all pseudonyms.

## Findings

Overall, the participants largely perceived data visualisations as trustworthy, with the majority rating them at three or above on the trustworthiness scale. Only the two data visualisations based on survey data with unknown data sources were rated below three by approximately one third of the participants. The types of data visualisations did not appear to affect the participants’ perception. What mattered was the way data was presented, the data sources and the news media. Data visualisations with clear data sources and published by broadsheets were rated higher for trustworthiness than those lacking data source information, those with data sources unknown to the participants, or the one produced by the tabloid.

Likability and learnability were not corresponding to trustworthiness. For example, the data visualisation by *the Daily Telegraph* regarding following government advice received low trustworthiness scores due to unclear survey data and a data source unfamiliar to participants; however, it received high likability scores. By contrast, *The Economist*’s data visualisation about flattening the curve was rated low for likability but had high trustworthiness scores. The following sections will outline the main aspects of participants’ perception of these data visualisations’ trustworthiness and related influencing factors.

### The influence of data visualisations' presentation

The 11 data visualisations include six types: line graph, bar graph, map, scatter plot, interactive and inforgraphic. However, participants did not mention the types of data visualisations as a factor when discussing trustworthiness. Their trustworthiness ratings also demonstrate the insignificance of data visualisations’ types, as no specific type received particularly low or high scores, except for a bar graph and an infographic that had slightly lower scores than others because they used survey data and unknown data sources. However, three aspects of data visualisation presentation: problematic presentation, presenting obvious intention and presenting data out of context influenced participants’ perception of trustworthiness.

Firstly, problematic presentation, including issues with bar and line presentation, colour choices and wording, undermined trust. A participant from a medical background expressed confusion regarding the inappropriate use of bars to present multiple sets of data in a bar graph:

*“I think it is trying to match the data in all these, but one (bar) is based on 6 per 10,000, the other is based on incidents of 20 per 10,000, but they are trying to put that information (together), trying to make it equal. … It is not the same comparison, but (what) they are trying to show is (to compare) all three of them … But, ... it is not actually compatible, ... it can be portrayed as giving the wrong message to the general public.” (Jess, a medical professional)(content in parentheses was added by the author)*

Likewise, a participant from an administration background also voiced concerns about the problematic presentation of an infographic featuring multiple line graphs:

*“I don’t think the graphs are a very good visual representation of the numbers. How is that, that says 41.3, the same length (with that showing 14.1)? … Do you see what I mean? Each of those graphs looks the same but the numbers are quite different.” (Monica, an administrator)*

The problematic presentation also referred to mistakes, exemplified in the following quote:

*“The spelling mistake (in the data visualisation) really makes me very uncomfortable about the quality of this graph” (Vince, a university student studying politics)*

Secondly, the obvious viewpoints and intentions presented in data visualisations weakened the trust of the participants. Examples of this include:

*“Let’s say, trustable, three. …. It looks, to me, like that’s a graphic that’s been used to demonstrate a point, which is probably, *‘*Look at America, it’s so dreadful, they haven’t sorted it out, we hate Trump.*'* or something like that. … I think they’re using it just to support an angle.” (Monica, an administrator)*

*“Well, I think the graph is portraying an intention, so it’s not saying, ‘* *This is what we’ve measured.' It’s saying,* *‘This is what we are trying to do.'”* *(Rob, a software engineer)*

Thirdly, participants commonly cited the presentation of data without proper contextualisation as a significant factor diminishing their trust. They would like to see data was visualised in its context or brief information was included in the visual representation. For example, several participants reduced their ratings for *The Guardia*n’s line graph representing daily case numbers since 2020, as the graph did not provide the context of different testing scales in different times in the UK, as exemplified in this quote:

*“I don’t think it can be trusted that much, especially, you know, in the first lockdown, we didn’t really have the tests and stuff. So, … this may not actually represent the truthful information. So, I would put a note saying that that was the case, and I’m thinking, if someone wanted to look at this chart in 30 years’ time, that wouldn’t be very informative for them because they wouldn’t know this” (Nancy, a data analyst).*

This observation may be attributed to the absence of accompanying text, where this particular piece of information could have been found.

A participant with a marketing background also recognised the importance of context in relation to the representation of COVID-19 case numbers, where the size of population should have been taken into consideration:

*“And the thing with the number of cases, I suppose, is you don’t necessarily get told how many people are living there. … For complete transparency, what you’d need to do is to find a way of showing how many people were affected. If it’s 600,000, then what is that per head of population? ”(Emma, a marketing professional)*

### The perceived transparency and trustworthiness of data sources

The perceived transparency and trustworthiness of data sources were identified as common factors that influenced participants’ perception of the trustworthiness of data visualisations. Most participants referred to the data source while explaining why they (dis)trusted the data visualisations. Data visualisations that lacked this information usually received low scores for trustworthiness, as shown in the following example:

*“Trust? Well, I don't know the source. Where is this (data) from? … I would say 2.5 out of 5.” (Sam, a Master’s student studying architecture)*

Although most participants admitted they would not check and verify the data, seeing the link to the data source and information about the data source provided reassurance:

*“Although as a resident of the country who just wants to know how much trouble we are in with coronavirus, I don't download that data and look at it. But it’s important that it is there.” (Rob, a software engineer).*

This means that including data sources and related links in data visualisations is symbolic rather than practical and may not effectively encourage audiences to use open data for societal benefits.

However, the extent to which including information about data sources can enhance the trustworthiness of the data visualisations depended on participants’ familiarity with and trust in them. Data sources such as the “Centre for Disease Control and Prevention” and “coronavirus.data.gov.uk”, were thought to be more trustworthy than data sources such as “JL Partners” and “SAVANTA”. As the latter sources were unknown to participants, they were perceived as untrustworthy. For example, a participant said:

*“I don’t know who JL partners is … it doesn’t strike me as something which has reliability” (Vince, a university student studying politics)*

Even participants with data or IT backgrounds were unfamiliar with “JL Partners” and “SAVANTA” and perceived them as undermining the trustworthiness of the data visualisations.

While most participants regarded government sources such as data.gov.uk as trustworthy, a few expressed scepticism due to their lack of trust in the government. For instance, a participant with an IT background provided a detailed explanation of his scepticism:

*“Remember, this is the same government that when the numbers were not looking in their favour, they were doing this regular reporting, every day they were going out there in the press and they were getting hammered. The press, the media, people like us, the public, lost faith in them because they were getting hammered. What was their response? They stopped doing daily briefings then.”*

*…*

*“When you see that sort of stuff happening, you then go, ‘Okay, how confident should I be?' Again, it’s one of those cases where you can literally turn round and go, ‘Okay, the government is saying this.' I would start looking at the source and go, ‘Okay, well where are they getting it from and what’s sitting behind them.’* *Or I’d look at this information and go and get a more qualified opinion.” (Jack, an IT project manager)*

The concerns of participants sceptical about government data sources were also derived from their scepticism about data production, particularly among those possessing substantial knowledge about data. This will be discussed in the next section.

### Scepticism about data production

Participants, especially those whose work closely involved data and thus possessed higher levels of data literacy, expressed scepticism regarding data production, which emerged as a main factor impeding their perceived trustworthiness of data visualisations. They believed the trustworthiness of data visualisations did not come from journalists’ intention to handle the data carefully and truthfully represent them. Instead, they perceived it as contingent upon the trustworthiness of the underlying data, which, however, could be greatly influenced by what happened in the data production process. As audiences, their lack of knowledge of the influence on the data production process prevented them from fully trusting that data visualisations accurately represented reality. They suspected that even the journalists who created the data visualisations were uncertain about the trustworthiness of the data. Therefore, interestingly, participants made a distinction between the trustworthiness of data visualisations, which they believed originated from journalists’ intention to convey the truth, and the trustworthiness of the underlying data, which they perceived as beyond journalists’ control. A striking consensus among participants, particularly those with backgrounds in IT or data, was that the trustworthiness of data visualisations was closely linked to the factors influencing the collection and production of the underlying data, such as how COVID cases were identified and recorded and how cases or people were classified in a survey. For example, a participant commented on the survey data used in a data visualisation:

*“How do you get the information? How do you classify the people? What’s the difference between the ‘Mostly Sometimes’ and ‘Mostly Not’? Where did you get the data from?*

*I don’t trust this information. Because I know it’s extremely difficult to get accurate information for this category” (Nancy, a data analyst).*

A participant with an IT background made a comment:

*“… there’s little consistency between the data sources (in different countries). And it’s a question mark if the authority is cooperative to publish the data in a transparent way. … I can trust that the visualisation showing what is contained in the data source. … But whether the data source is telling the truth or not, that’s a completely different story. …” (Henry, a data architect).*

Another participant with an IT background echoed this perspective:

*“… I think the data is less trustworthy because I know that all the countries are measuring things in different ways. … if you are asking how trustable it is, the question is only answerable by finding out where the data came from and how it was collected. We don’t know. It’s just a guess.” (Rob, a software engineer).*

Participants also showed a consensus about the criticality of data context in establishing the trustworthiness of both data and data visualisations. For example, a participant with an education background commented on a data visualisation that used survey data without including information about the polling company and the poll itself:

*“I think knowing who carries out a poll is important because different polling companies have different agendas. … it also doesn't say how big the sample was, which means that I can't really make a good judgement about it.” (Rachel, an education professional)*

This point connects to the earlier discussion on data visualisation presentation: it would be beneficial for standalone data visualisations to include brief information about the background of the data. Certainly, this background information could have been included in the text of the news articles, which participants were not be provided with.

### (Un)trustable because of the news media that published the data visualisations

Participants’ perception of COVID-19 data visualisations was also influenced by the perceived trustworthiness and reputation of the UK news media, which was weighed against their doubts about data sources. Some participants even viewed the trustworthiness of news media that published the data visualisation as more important than that of data sources.

Participants considered quality newspapers, particularly *the Economist*, *The Guardian* and *The Financial Times* and those that shared their perspectives to be trustworthy, while perceiving tabloids as less trustworthy. For example, one university student, who rated four out of five for one data visualisation, placed greater emphasis on the news outlet rather than the data source:

*“I can see at the bottom that it’s from The Economist, and that’s a fairly established news form, so I trust seeing it from there. If it was on, saying something like The Daily Mail or The Sun, I might question it a bit more, because they tend to be more biased. And they do post a lot of gossip kinds of things, so I don’t always fully take what they say as the truth.” (Phoebe, a university student studying psychology)*

Likewise, two participants with a marketing background said:

*“I mean if it was The Economist that’s publishing it, then, for me, I would probably trust it. It’s just because I would expect them to know what they’re publishing there and what statement they’re making there.” (Rose, a marketing professional).*

*“Okay. I do tend to-I shouldn’t really. I do tend to trust what I read in The Guardian and The Independent. … I don’t deny they have a political agenda but it feels like maybe, I guess, their perspective is more aligned with mine.” (Emma, a marketing professional)*

Most participants expressed distrust in *the Daily Mail*—the only tabloid included in this study, as shown in the following quote:

*“Because obviously I don’t trust the Daily Mail. They have a history of showing lots of bias on different topics, on different things. They’re very forceful and try to push people to their view and what they want the people to follow, go by what they want them to go by. I don’t know how much I can trust this data because it’s from the Daily Mail.” (Jacob, a university study studying engineering)*

In cases where data sources were absent from the data visualisations, the trustworthiness of the news media played a role in enhancing participants’ trust in the data visualisations. For example:

*“(There is no data source.) I suppose I am biased as well, because I think the FT wouldn’t publish something that didn’t have a proper source. It is naughty that they didn't put the source (in the data visualisation), but it may be in the article somewhere. It probably is.” (Rachel, an education professional)*

### Lived experiences, pre-gained information and pre-existing understanding

Participants frequently relied on real-life lived experiences during the COVID-19 pandemic and information from other sources as reference points when assessing the trustworthiness of the data visualisations. Often, participants chose to place trust in a data visualisation when the information presented in the data visualisation was aligned with their existing understanding of the situation:

*“It makes sense to me and it kind of does fit in line with what I know from last year, so I would probably trust it.” (Phoebe, a university student studying psychology).*

*“*... *because it supports, anecdotally, the information I’m seeing from other sources, I’m prepared to give it a 3.” (Jack, an IT project manager)*

*“Because, again, it matches my own personal view of how I probably acted within the pandemic. To what extent if it’s, kind of, matching what I’ve actually been doing myself.” (Rose, a marketing professional)*

Medical professional participants frequently turned to their knowledge about the situation gained from their work to verify how truthful the data visualisations were. While evaluating the *FT* data visualisation about the official death toll only telling part of the story, Jess, a medical professional, made the following comments:

*“It is because I think I was aware this information was shared as well about these COVID-19 deaths and not reporting (all COVID-19 deaths), or this mismatch of information as well. That was discussed in some of our forums as well in the beginning. So, maybe that is why.”*

Another very interesting point is that participants frequently attempted to compare the information they perceived from the data visualisations with what they observed in the news to form a judgement. Examples include:

*“Yes, yes, I trust it because I think it is corroborated by other things that I have seen. I mean I have heard this concept said by lots of scientists and epidemiologists, so I would agree with that, yes.” (Rachel, an education professional)*

*“Looking at it now, I would say five, because it does simply replicate what has been in the news.” (Jill, a medical professional)*

On occasions where the data visualisations did not match participants’ understanding gained from lived experience or information they already had, they tended to regard data visualisations as less trustworthy. For example, a participant explained why she did not entirely trust a data visualisation, which showed that some countries, such as Indonesia and Malaysia, had lower case numbers than others, such as the US:

*“A country like Indonesia or Malaysia, they reported a certain number of cases but actually I heard from the people who lived there, the actual situation is worse than what being reported because some cases in a bit rural area are not reported.” (Charlotte, an education professional).*

## Discussion and conclusion

**Figure 1.**
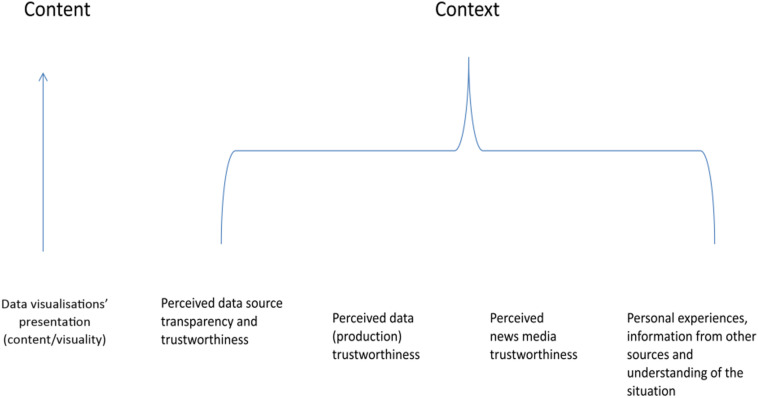
Influencing factors identified in the study.

The above discussion of audience perception of the trustworthiness of COVID-19 data visualisations identifies five main influencing factors: (1) data visualisations' presentation (content/visuality), (2) perceived data source transparency and trustworthiness, (3) perceived data (production) trustworthiness, (4) perceived news media trustworthiness, and (5) personal experiences, information from other sources and understanding of the situation (see Figure 1).

These factors highlight the important role social context plays in influencing audience perception of the trustworthiness of data visualisations. Participants’ perception was not only influenced by the content itself, including how data was presented in data visualisations (content/visuality), which is similar to the concept of “message credibility” (Hovland, et al., 1953; O'Keefe, 1990). It was also—to a greater extent - influenced by the context, in which they gained their pre-existing understanding of the data, the data sources, the news media and the overall situation. Therefore, their perception was significantly affected by factors extending beyond the realm of data visualisations and into social context.

The existing literature has recognised the importance of social context to audience perception of news trustworthiness. But by social context, these studies mainly mean external factors, such as audiences’ backgrounds such as numeracy levels (Gondwe, et al., 2021), their motivation and incentive to process information and prior knowledge (Lee and Kim, 2016), political stances and values, partisanship (Kohut and Toth, 1998; Horowitz, et al., 2021) and political and personal cynicism (Lee, 2010). While confirming the significance of participants’ backgrounds, this study takes these discussions further by identifying the influence of three-leveled social context. The three levels of social context indicate the critical role played by data—a socially constructed product—in influencing the perception of audiences regarding the trustworthiness of data visualisations.

The first level presents the social context where data is collected, produced, curated and archived. Participants’ trust (or mistrust) in data visualisations was influenced by their varied understandings of data as a socially constructed entity shaped by social factors throughout the production process.

The second level of social context is related to where data is retrieved—the data source. This is similar to what Hovland and his collaborators described as “source credibility” (Hovland, et al., 1953), although their term merely refers to the trustworthiness of the communicator. The trustworthiness of data sources refers not only to their own trustworthiness but also to how the data is produced, which occurs at the first level of social context.

Social context also means the social context in which audiences live and gain information and understanding about the situation. In this study, participants' lived experiences and the information they received in the past from various sources shaped their understanding of the situation. These anecdotal experiences, cognition, and frames of reference gained from their living environment greatly influenced their perception of data visualisations’ trustworthiness.

The role of social context thus highlights the importance of participants’ backgrounds, particularly their job and personal experiences, to their perception of data visualisations’ trustworthiness. These backgrounds enabled them to develop a specific understanding of data and COVID-related reality, which they relied on to make judgements about data visualisations’ trustworthiness. The trustworthiness of data visualisations was not solely dependent on their content or presentation, although these aspects were important. Instead, it extended to the social context associated with data visualisations and their audiences. It was up to how participants perceived the trustworthiness of the data sources and the news media and their knowledge of data and data production. Whether the meanings of data visualisations matched their understanding of the situation gained from previous lived experiences and information received from other sources was also vital. These suggest that audiences’ lived experiences and the information they receive from various sources become significant reference points when they assess the trustworthiness of data visualisations related to events such as the COVID-19 pandemic, which have implications for people’s personal and, in some cases, professional lives. The social construction nature of data and data visualisations opens a space for audiences to question data visualisations’ trustworthiness, which is thus beyond the control of journalists and news media. Hence, data visualisations should not be viewed merely as graphs but rather as cultural products of social context.

Traditionally, data visualisations have been thought to be a useful journalistic tool to tell news stories and engage audiences (Flew et al., 2010; Ojo and Heravi, 2018; Stalph and Heravi, 2021). In this study, participants largely regarded data visualisations trustworthy, which confirms their usefulness to boost audience trust in news, in particular in the UK context where trust in the news is low. However, this study also suggests audiences may take into account aspects of data visualisations that differ from those considered by journalists. The importance of social context suggests that data visualisations, as cultural products of social context, may have a precarious ability to enhance audiences’ trust in news media, at least if they are considered in isolation from the accompanying text of news articles. This applies not only to news media but also to other knowledge institutions that use data visualisations to convey messages to the public. The strong connection between trust in data visualisations and trust in data, a socially constructed product, suggests that audiences with higher levels of data literacy would be more likely to question the credibility of data visualisations based on their knowledge of data. However, for audiences with lower levels of data literacy and limited awareness of the uncertain nature of data, data visualisations can be deceptively credible, leading them to unquestioningly accept the reality represented in data visualisations as the true reality. Therefore, efforts to improve their data literacy are necessary to enable them to have a more appropriate appreciation of data visualisations.

This study contributes to our understanding of audience perception of data visualisations’ trustworthiness. However, it has limitations due to the small sample size, the participants’ high levels of education and data literacy, the absence of accompanying text for the data visualisations, and the fact that the majority of the data visualisations were taken from quality newspapers. The findings did not confirm emotional reading of participants as found by other scholars (Kennedy and Hill, 2018; Sleigh and Vayena, 2021). Instead, the interviews were full of rational discussions and judgement. This could be attributed to the participants being well educated and having professional backgrounds. The findings, such as the significance of participants’ lived experiences and pre-existing knowledge, may have been influenced by the fact that COVID-19 data visualisations are about the topics that they have personally experienced. In addition, the examination of data visualisations in isolation from the text of news articles may have been a reason why participants regarded data visualisations lacking context.

For further research, it would be beneficial to involve participants from diverse social backgrounds and data visualisations published by tabloids or broadcasters to gain a comprehensive understanding of how participants’ backgrounds and types of news media may influence audience perception of data visualisations’ trustworthiness. It would also helpful to explore whether audiences perceive data visualisations’ trustworthiness in a similar manner in social contexts outside of the UK, considering that COVID-19 management and the public-press relationship may have characteristics specific to each context. This research also implies the importance of the immediate reading context of data visualisations, which is, however, removed in this study. Future research may aim to investigate the influence of including the text of news articles on audiences’ trust in data visualisations.
